# Male Army ROTC Cadets Fail to Meet Military Dietary Reference Intakes and Exhibit a High Prevalence of Low Energy Availability and Poor Sleep Quality

**DOI:** 10.3390/jfmk8030095

**Published:** 2023-07-05

**Authors:** Taylor Garron, Dylan J. Klein

**Affiliations:** Department of Health and Exercise Science, Rowan University, Glassboro, NJ 08028, USA

**Keywords:** tactical athlete, sports nutrition, sleep quality

## Abstract

The purpose of this study was to assess the dietary habits, prevalence of low energy availability (EA), and sleep quality in a cohort of male army Reserve Officer Training Corps (ROTC) cadets, and to investigate the relationship between EA and sleep quality as well as EA and various body composition variables that are important for tactical readiness. Thirteen male army ROTC cadets (22.2 ± 4.1 yrs; BMI: 26.1 ± 2.3) had their EA and body composition assessed using diet and exercise records alongside bioelectrical impedance analysis. Cadets also completed a validated sleep questionnaire. Sixty-two percent of participants presented with clinically low EA (<30 kcal/kg fat-free mass [FFM]) and none met the optimum EA threshold (≥45 kcal/kg FFM). Dietary analysis indicated that 15%, 23%, 46%, 23%, and 7% of cadets met the Military Dietary Reference Intakes (MDRI) for calories, carbohydrates, protein, fat, and fiber, respectively. Additionally, 85% of cadets exhibited poor sleep quality. Significant associations between EA and fat mass/percent body fat were shown (*p* < 0.05). There was, however, no statistically significant correlation between EA and sleep quality. The present study found a high prevalence of low EA and sleep disturbance among male army ROTC cadets and that many were unable to meet the MDRIs for energy and macronutrient intake. Further, low EA was associated with higher percent body fat and fat mass but not sleep quality.

## 1. Introduction

The United States (U.S.) army recognizes nutrition and sleep as critical behavioral modifications to optimize tactical readiness and mission success [1]. As elite tactical athletes, professional soldiers must be ready to defend the nation in the face of demanding physical, emotional, and cognitive challenges. If performance cannot be optimized, national security and safety are at risk. Therefore, meeting military-recommended guidelines for nutrition and sleep is of utmost importance.

Soldiers are often confronted with periods of sustained energy and sleep deprivation. Such prolonged periods can pose major health and tactical readiness concerns. Relative energy deficiency in sport (RED-S) is the culmination of such concerns, describing a condition characterized by disrupted metabolic and endocrine function, increased injury risk, and the potential for athletic performance loss [2,3]. A primary factor underpinning RED-S is low energy availability. Energy availability (EA) is defined as the amount of dietary energy available to support physiological function (e.g., reproductive and endocrine functions) after accounting for the energetic cost of exercise [4]. This equates to the difference between energy intake (EI) and exercise energy expenditure (EEE), normalized to fat-free mass (FFM). Sustained low EA (i.e., <30 kcal/kg FFM) has been shown to depress levels of key hormones such as luteinizing hormone (LH), estrogen, triiodothyronine (T3), insulin-like growth factor-1 (IGF-1), and leptin, alongside increases in cortisol and various bone resorption markers leading to poor metabolic health and reduced bone mineral density [5]. Additionally, chronic low EA can potentially lead to reduced performance and increased injury risk [6], thus placing soldiers at a tactical disadvantage. Accordingly, low EA poses a large concern for the soldier seeking to optimize tactical readiness and to manage mission success.

The Reserve Officer Training Corps (ROTC) cadets are a unique subset of the military population given that they are a mixture of professional soldiers and college students. As such, these cadets must navigate the physical and cognitive demands of military training alongside academic and extracurricular responsibilities. Consequently, these cadets are at risk for poor nutrition and sleep. Previous studies in college athletes, who also must maintain high levels of athletic and academic success, have commonly shown nutritional intakes below sport-specific recommendations [7,8,9] alongside a high prevalence of low EA, especially in endurance athletes [7,8,9,10,11,12]. Sleep quality and duration have also been shown to be inadequate in college athletes, leading to greater daytime sleepiness and the inability to stay awake for daily activities [13]. Likewise, ROTC cadets have been shown to exhibit poor sleep quality, sleep duration, and increased daytime sleepiness [14]. Recent research in athletic trainers has even shown a higher relative risk (RR = 1.146) of sleep disturbance in those who present with low EA [15], suggesting a link between these two factors. To date, however, no such studies have concomitantly looked at, or investigated the relationship between, EA, and sleep quality in ROTC cadets.

Given the lack of observational data in this population, the purpose of the present study was to assess the dietary habits, prevalence of low EA, and quality of sleep in a cohort of army ROTC cadets. We also aimed to investigate the relationship between EA and sleep quality as well as EA and various body composition variables that are important for tactical readiness.

## 2. Materials and Methods

### 2.1. Participants

The Rowan University Institutional Review Board (IRB) approved this study. Forty-five army ROTC cadets were briefed on the study and its components. Twenty cadets voluntarily agreed to participate in this study by providing written informed consent. Only 13 males fully completed dietary, exercise, and body composition analyses. Data were collected during the start of the Spring academic semester. Cadet characteristics are located in Table 1.

### 2.2. Dietary Intake

Dietary habits (i.e., breakfast, lunch, dinner, and snack consumption) as well as energy and macronutrient intakes of participants using a self-reported 3-day diet record. In brief, each cadet was instructed on how to keep a 3-day diet record and we provided them with a detailed instruction manual for them to reference. Participants recorded their usual diets over the course of one week by providing dietary information from two, non-consecutive weekdays and one weekend day. Upon return, each diet record was reviewed together with the participant for completeness. Additional information (e.g., meal type, brand names, volume/size measurements, and cooking methods) was added to the diet record when appropriate. Energy (kcal/d) and macronutrient intake (g/d) were analyzed using ESHA Food Processor (Salem, OR) professional dietary software (version 11.7). Relative energy and macronutrient intakes were normalized to body mass (g/kg/d).

### 2.3. Exercise Energy Expenditure

We estimated EEE using a 7-day exercise record for all participants. Exercise records included all structured physical activity conducted including modality, duration, and intensity. Records were reviewed upon return for completeness. Exercise records were then compared to The Compendium of Physical Activities to calculate metabolic equivalents (METs) for each type of exercise activity. We used the following equation to calculate EEE:

EEE = body mass (kg) × time (h) × METs (1 kcal/kg/h).

### 2.4. Body Composition

Body composition (body mass [BM], fat mass [FM], fat-free mass [FFM], percent body fat [%BF], and skeletal muscle mass [SMM]) was assessed using the InBody 770 multi-frequency bioelectric impedance analysis (BIA) unit (Cerritos, CA, USA). All measurements were conducted between 7:00–10:00 a.m. in a fasted state (i.e., no food 8–9 h prior to testing).

### 2.5. Energy Availability

Using EI, EEE, and FFM data, we calculated EA using the following equation:

EA = (energy intake [EI] − exercise energy expenditure [EEE])/fat-free mass (FFM).

Energy availability categories were defined as follows: optimal EA (≥45 kcal/kg FFM/d), sub-optimal EA (30–44 kcal/kg FFM/d), and clinically low EA (<30 kcal/kg/FFM/d) [16].

### 2.6. Pittsburgh Sleep Quality Index

All participants completed the Pittsburgh Sleep Quality Index (PSQI) questionnaire. The 19-item questionnaire assesses sleep quality and sleep disturbances over the previous 30 days. It asks participants to self-report their frequency of daytime and nighttime sleep dysfunction, medication use, and sleep duration. The 19 self-rated items are combined to form seven “component” scores, each of which has a range of 0–3 points. The seven components are as follows: subjective sleep quality, sleep latency, sleep duration, habitual sleep efficiency, sleep disturbances, use of sleeping medication, and daytime dysfunction. The seven components scores are then added to yield a “global” score. A score ≥5 indicates disturbed sleep within the past month. The PSQI has a reported sensitivity of 89.6% and a specificity of 86.5% with a Cronbach’s alpha of 0.83 [17].

### 2.7. Statistical Analysis

Participants’ demographics, dietary, and sleep quality information is presented using descriptive statistics (means ± standard deviations [SD]). Differences between cadets with and without clinically low EA were carried out using the student’s *t*-test. Pearson correlation coefficients were used to examine the relationships between EA/EI and body composition variables and EA and sleep quality scores. Correlations were interpreted using the following criteria: very weak: <0.20; weak: 0.20–0.39; moderate: 0.40–0.59; strong: 0.60–0.79; very strong: >0.80 [18]. One-way analysis of variance (ANOVA) was used to compare EI across meals (breakfast, lunch, and dinner). Macronutrient intake values were compared to the most recent Military Dietary Reference Intakes (MDRI) as set forth by the Departments of the Army, Navy, and Air Force as part of the Nutrition and Menu Standards for Human Performance Optimization [19]. All statistical analyses were conducted using GraphPad Prism software version 9.5.1 for Windows (San Diego, CA, USA). Significance for all tests was set at an alpha of ≤0.05.

## 3. Results

### 3.1. Participants

Cadets’ demographic and body composition characteristics can be found in Table 1.

### 3.2. Energy Intake, Exercise Energy Expenditure, and Energy Availability Prevalence in Male Army ROTC Cadets

**Figure 1:**
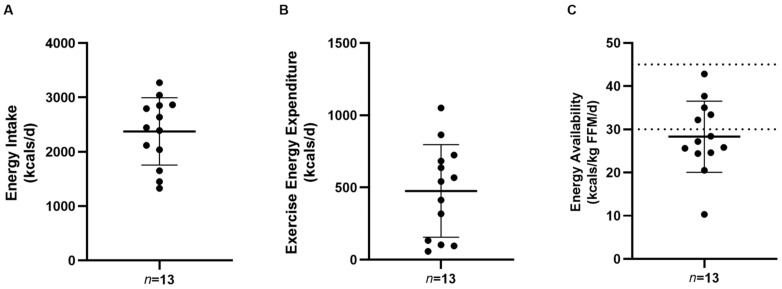
Energy intake, exercise energy expenditure, and energy availability in male army ROTC cadets (*n* = 13). Values are reported as means ± SD (**A**–**C**). Optimal (≥45 kcal/kg FFM), sub-optimal (30–44 kcal/kg FFM), and clinically low (<30 kcal/kg FFM) EA cut-offs are indicated by dashed lines (**C**).

Cadets’ EI, EEE, and EA data are displayed in Figure 1. Mean EI, EEE, and EA among the cadets was 2375.4 ± 620.7 kcal/d, 476.2 ± 320.1 kcal/d, and 28.3 ± 8.2 kcal/kg FFM, respectively. No cadet achieved optimum EA (>45 kcal/kg FFM), whereas 38% (*n* = 5) of cadets were found to have sub-optimal EA (30–44 kcal/kg FFM) and 62% (*n* = 8) of cadets were identified as having clinically low EA (<30 kcal/kg FFM).

### 3.3. Energy and Macronutrient Intakes Relative to the Military Dietary Reference Intakes (MDRI) in Male Army ROTC Cadets

**Figure 2:**
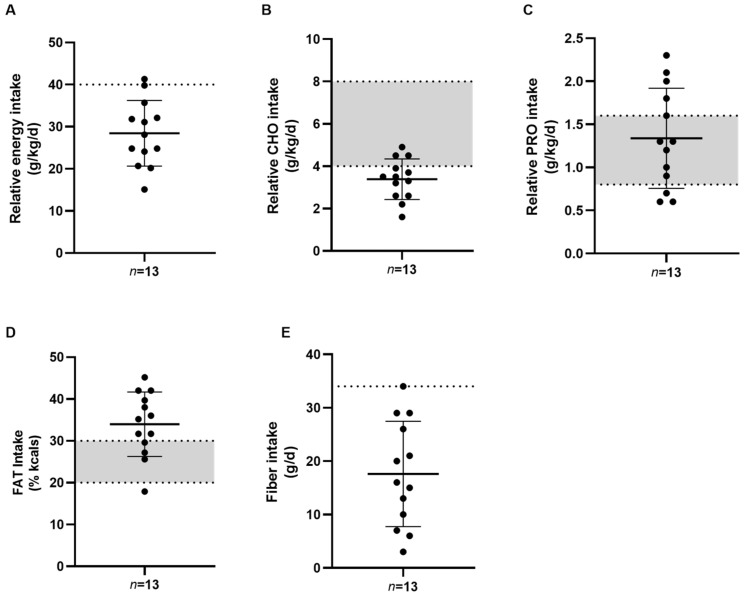
Relative energy and macronutrient intakes in male army ROTC cadets (*n* = 13). Values are reported as means ± SD. The Military Dietary Reference Intake (MDRI) for relative EI (40 kcal/kg) is represented by a dashed line (**A**). Grey shaded areas (**B**–**D**) represent the MDRI ranges for CHO (4–8 g/kg/d), PRO (0.8–1.6 g/kg/d), and FAT (20–30% kcals). The MDRI for fiber for males (34 g/d) is represented by a dashed line (**E**). CHO = carbohydrate; FAT = fat; FFM = fat-free mass; PRO = protein.

Mean relative energy and macronutrient intakes are displayed in Figure 2. Mean relative EI was 28.4 ± 7.8 kcal/kg and only one cadet met the MDRI threshold of 40 kcal/kg (Figure 2A). Mean relative carbohydrate and protein intake was 3.4 ± 0.9 g/kg and 1.3 ± 0.6 g/kg, with 23% (*n* = 3) and 46% (*n* = 6) of cadets meeting the MDRI, respectively (Figure 2B,C). Mean fat intake as a percentage of total calories was 34.0 ± 7.7% with all but one cadet meeting or surpassing the MDRI (Figure 2D). Mean fiber intake was 17.6 ± 9.9 g with only one cadet meeting the MDRI (Figure 2E).

### 3.4. Energy and Macronutrient Intakes by EA Status in Male Army ROTC Cadets

Energy and macronutrient intakes by EA status can be found in Table 2. Cadets categorized with clinically low EA had significantly more FM and %BF (*p* < 0.05) and ate significantly fewer kcals (absolute and relative intakes), carbohydrate (absolute and relative intakes), and fat (relative intake) (*p* < 0.05) than their non-low EA counterparts.

### 3.5. Dietary Habits of Male Army ROTC Cadets

Cadets’ meal consumption patterns can be found in Table 3. There were no significant differences between the mean energy content of the meals (*p* = 0.661). Of the 39 potential opportunities for breakfast, eight of these meals (20.5%) were skipped by the cadets. Similarly, nine lunch meals (29%) were also skipped, and only one dinner meal was not achieved by the cadets. Two cadets reported consuming one snack each, with the majority (*n* = 11) forgoing this opportunity altogether for energy and macronutrient intake.

### 3.6. Sleep Quality of Male Army ROTC Cadets

The Pittsburgh Sleep Quality Index analyzed seven components of sleep quality and disturbance in cadets. The mean global PSQI score was 6.8 ± 2.5 (range 3–11) with 85% of participants (*n* = 11) indicating varying levels of sleep disturbance and poor sleep quality (global PSQI ≥ 5). Mean component scores for each section can be located in Table 4. Regarding total hours of sleep, the cadets achieved an average of 6.0 ± 1.0 h per night.

### 3.7. Correlational Analyses

Correlational analyses between EA, EI, and body composition variables are shown in Table 5. Significant (*p* < 0.05) relationships between EA and %BF and FM, as well as EI and %BF and FM, were seen. There was no statistically significant relationship between EA and sleep (r = −0.300, *p* = 0.320).

## 4. Discussion

To the best of our knowledge, the current study is the first to concomitantly assess the dietary habits, prevalence of low EA, and sleep quality in a cohort of college, male army ROTC cadets. These data further the literature on EA and sleep in the military population and extend what little is known about EA in males. The primary findings show that the mean EA of male army ROTC cadets was 28.3 ± 8.2 kcal/kg FFM and the prevalence of clinically low EA to be 62%. This is similar to a study by Edwards et al. who also reported 69–92% low EA prevalence among 13 British army Officer cadets during campus, field exercise, and camp/field training periods [20]. These findings are also in agreement with what has been reported in college athletic populations [7,9]. For example, the prevalence of low EA in college endurance athletes range from 20–67% [8], with most studies; however, examining EA in female athletes rather than males. Given this fact, it should be noted that there is debate regarding the threshold for clinically low EA in males. Recent research indicates a more severe EA restriction level may be necessary (i.e., <15 kcal/kg FFM) to induce the negative endocrine and metabolic consequences typically seen in female athletes (e.g., poor bone mineralization and disruptions in reproductive function) [21]. As such, more research analyzing the prevalence of low EA and its long-term consequences in male cadets and soldiers is highly warranted.

A well-chosen diet is critical to optimizing performance and training adaptations [22]. Nutritional analysis indicated that the cadets were not meeting many of the energy and macronutrient recommendations as per the MDRIs. The mean EI was 2375 ± 620.7 kcal/d, which is similar to a report by Daniels et al. who also showed an average EI of 2414 kcal/d in male army ROTC cadets [23]. This is well below the military’s recommendation of 3400 kcal/d for the average 85 kg male doing moderate levels of activity [19]. When normalized to BM, only two cadets in the present study met the military’s recommendation of 40 kcal/kg, highlighting the potential need for greater EI in this population. Further, a large percentage of cadets failed to meet the carbohydrate and protein MDRIs of 4–8 g/kg and 0.8–1.6 g/kg, respectively. This is partly in contrast to a study by Lutz et al. [24] who evaluated the dietary intakes of basic combat training recruits and showed adequate intake of calories, carbohydrates, and protein relative to the MDRIs. Inadequate energy and macronutrient intake are, however, consistent across studies on college athletes [7,12,25] indicating a similarity between college ROTC cadets and college athletes. This, however, is not surprising given the physical demands and academic and extracurricular time commitments that overlap these populations.

When differentiated by EA status, cadets with clinically low EA were shown to have lower intakes of calories (both absolute and relative intakes), carbohydrates (both absolute and relative intakes), and fat (relative intake) than their non-low EA peers. Previous studies have shown that athletes with low EA more commonly report consuming fewer calories and carbohydrates than athletes without low EA [26,27]. Similarly, Magee et al. [9] also reported lower relative fat intake in NCAA Division III female soccer players with low EA compared to those without low EA. As a result, reduced intakes of energy and key macronutrients could have negative performance implications for cadets, especially during intensive training activities. To that end, while the effects of EA, per se, on military performance have not been studied, a review by O’Leary and company [28] highlights the performance decrements seen with energy deficits in various military settings. For example, muscle strength and power [29,30], occupational task performance [31], and endurance capacity [32] have all shown decreases with varying levels of calorie deficit during military training operations. Further, a recent meta-regression analysis shows a loss in lower body muscle performance in proportion to the energy deficit in military personnel [33]. As such, it is clear that energy status is a contributing factor to tactical performance, but future studies need to look at the effects of EA, in particular, on performance in soldiers.

Interestingly, cadets with low EA were shown to have higher %BF and FM than those without low EA. While paradoxical, these results could indicate the initial stages of purposeful, restrictive eating practices in cadets with body compositions not commensurate with body image goals and a military physique/standard. In this vein, a previous study in ROTC cadets has shown a high prevalence of disordered eating risk and body image dissatisfaction, particularly among army ROTC cadets [34]. As it pertains to the current data set, we can only speculate as to whether these factors play a role in reduced EI and low EA. Alternatively, the dietary reporting literature has shown underreporting in participants with higher BMIs and bodyweight [35,36] and thus presumably higher FM and %BF. Despite no significant difference in BMI between cadets with and without low EA (*p* = 0.138, cadets with low EA did have higher %BF and a BMI indicative of overweight (≥25), whereas those without low EA had lower %BF and a BMI within the normal bodyweight range (18–24.9). As it stands, we cannot rule out underreporting as a function of weight status as a potential factor influencing the EA data.

In the present study, we also showed a high rate of skipping breakfast (i.e., 20.5% of potential breakfast meals) and lunch (i.e., 29% of potential lunch meals). Not only do these meal omissions prevent cadets from potentially meeting energy and macronutrient recommendations, but it may have negative physical performance impacts as well. Indeed, a recent study by Jayne et al. [37] highlighted various dietary behaviors that are associated with Army Physical Fitness Test (now known as the Army Combat Fitness Test; ACFT) performance and showed that soldiers who reported skipping breakfast had 2.43 lower odds of reporting high ACFT performance. In a similar vein, in over 13,000 army soldiers, Purvis and colleagues [38] showed that regular breakfast consumption (i.e., six times/week) was associated with healthier eating scores and that those with higher healthy eating scores were more likely to perform in the highest quartile on ACFT testing. Given that ROTC cadet career placement is dependent on ACFT performance, focusing on breakfast consumption may be a viable strategy, not only to increase overall EI and thus reduce the risk of low EA and RED-S but to optimize physical fitness performance and career trajectory.

Sleep is a critical component of optimal physiological function and physical recovery. Sleep deprivation and chronic poor sleep have been shown to negatively impact cognitive functioning [39], learning and memory [40], metabolism and endocrine function [41], and physical performance [42,43]. Reserve Officers’ Training Corps cadets are often required to wake up in the early morning for daily physical training, followed by their typical school courses and extracurricular activities. This places a large emphasis on sleep quality in these individuals for maintaining performance, mental focus, and academic success. As highlighted by the U.S. Army’s Performance Triad Guide [1], sleep is considered an essential component of mission success and optimal health. The results of this study showed that 85% of cadets experienced some level of sleep disturbance and decreased sleep quality. Further, these cadets failed to meet the 7–8 h of sleep recommended by the U.S. army [1]. Research suggests multiple nights of advanced waking or sleep restriction can negatively impact muscular strength and the hormonal response to exercise [44]. Additionally, in one study by Ritland et al. [14], it was shown that longer sleep duration in ROTC cadets correlated to increased motivation and cognitive processing performance. In a follow-up study, the same group extended the sleep time of ROTC cadets by 1.5 h and showed improvements in reaction time, athletic performance, and motivation [45]. Taken together, sleep is a critical component of physical and cognitive performance and college ROTC cadets represent a population in need of strategies to optimize sleep.

### Limitations

It is important to highlight that this study is not without limitations. Indeed, the risk of under-reporting dietary intake is always a concern with self-reported data. As such, there is a potential bias to overrepresent low EA [46]. Similarly, accurately estimating EA is limited by the self-reported nature of the EEE data and the failure to capture other modes of activity outside of structured exercise. Techniques such as doubly labeled water and activity monitors could enhance these measures, as well as the resting metabolic rate (RMR) ratio (RMR_ratio_) has been shown to be a better objective surrogate for low EA status [47]. A third limitation of the study is that it relied on BIA as a method for FFM determination. Previous studies have shown BIA to overestimate FFM relative to DEXA [48,49], which may also bias the low EA data. Finally, future research should analyze changes in EA over time and its impact on physical performance and sleep in cadets. The current study reflects a single time point in a relatively small sample size of college ROTC cadets. As a result, the findings may not be representative of all ROTC cadets or other military personnel, and it remains unclear the impacts of chronic low EA on metabolic and performance outcomes in this population.

## 5. Conclusions

The results of the current study suggest that there may be a high prevalence of low EA among army ROTC cadets but that these findings need replication. Additionally, these cadets may not consume enough calories, carbohydrates, or fiber relative to the MDRIs placing them at odds with MDRI and army recommendations to optimize tactical readiness. While the majority of cadets did not meet the recommended hours of sleep and reported some level of sleep disturbance in the previous 30 days, this was not related to EA. Cadets with low EA also had higher FM and %BF levels than their non-low EA peers which requires future investigation into the nature of this association.

## Figures and Tables

**Table 1:** Cadets’ characteristics.

Characteristic	Cadets (*n* = 13 Males)
Age (yrs.)	22.2 ± 4.1
Height (cm)	179.6 ± 6.9
Body mass (kg)	84.2 ± 10.7
BMI (kg/m^2^)	26.1 ± 2.3
FM (kg)	17.1 ± 6.0
%BF	20.0 ± 6.0
FFM (kg)	67.2 ± 7.9
SMM (kg)	38.3 ± 4.7

Data presented as means ± SD. %BF = percent body fat; BMI = body mass index; FFM = fat-free mass; FM = fat mass; SMM = skeletal muscle mass.

**Table 2:** Daily energy and macronutrient intake between male army ROTC cadets with and without low EA.

	Low EA (*n* = 8)	Non-Low EA (*n* = 5)
BM (kg)	86.8 ± 11.2	80.2 ± 9.5
FM (kg)	19.6 ± 3.8	13.0 ± 7.0 *
%BF	22.7 ± 3.9	15.7 ± 6.7 *
FFM (kg)	67.2 ± 9.9	67.2 ± 3.8
EEE (kcal/d)	488.8 ± 324.6	456.1 ± 349.7
Energy availability (kcal/kg FFM)	23.3 ± 5.8	36.2 ± 4.2 ***
Energy intake (kcal/d)	2059.6 ± 557.4	2880.6 ± 306.1 **
Relative energy intake (kcal/kg/d)	23.6 ± 4.9	36.1 ± 4.3 ***
Carbohydrate intake (g/d)	257.6 ± 85.5	322.4 ± 33.7 *
Relative carbohydrate intake (g/kg/d)	3.0 ± 0.9	4.1 ± 0.6 ***
Protein intake (g/d)	102.6 ± 61.2	127.4 ± 25.6
Relative protein intake (g/kg/d)	1.1 ± 0.6	1.6 ± 0.5
Fat intake (g/d)	75.8 ± 30.8	112.0 ± 23.5 ^^^
Relative fat intake (g/kg/d)	0.9 ± 0.3	1.4 ± 0.3 ^**^
Fat intake (% kcals)	33.5 ± 9.1	34.8 ± 5.7

Data presented as means ± SD. BM = body mass; EEE = exercise energy expenditure; FFM = fat-free mass; EA = energy availability. ^^^ *p* = 0.079, trend toward significance based on student’s *t*-test. * *p* < 0.05, ** *p* = 0.019, *** *p* < 0.008, statistically significant from low EA based on student’s *t*-test.

**Table 3:** Cadets’ meal consumption patterns (*n* =13).

Meal	Reported Number	Kcals
Breakfast	31	597.3 ± 389.1
Breakfast skipped	8	
Lunch	30	710.0 ± 555.3
Lunch skipped	9	
Dinner	38	794.4 ± 670.8
Dinner skipped	1	
Snack	2	101.6 ± 300.7

Data presented as means ± SD.

**Table 4:** Measures of sleep quality in male army ROTC cadets (*n* =13).

Component	Range (Min, Max)	Score
Subjective sleep quality	(0, 2)	1.1 ± 0.5
Sleep latency	(0, 3)	1.7 ± 1.1
Sleep duration	(0, 3)	1.4 ± 0.9
Habitual sleep efficiency	(0, 1)	0.5 ± 0.5
Sleep disturbance	(0, 1)	0.9 ± 0.3
Use of sleeping medication	(0, 1)	0.1 ± 0.3
Daytime dysfunction	(1, 2)	1.2 ± 0.4
Global PSQI	(3, 11)	6.8 ± 2.5

Data presented as means ± SD.

**Table 5:** Relationships between body composition and energy variables in army ROTC cadets.

	Mean EA (kcal/kg FFM)	*p*-Value	Mean EI (kcal/d)	*p*-Value
BM (kg)	−0.333	0.267	−0.307	0.307
%BF	−0.578 *	0.039	−0.610 *	0.029
FFM (kg)	−0.031	0.919	0.0399	0.897
FM (kg)	−0.554 *	0.049	−0.602 *	0.030
SMM (kg)	−0.020	0.949	0.0396	0.897

%BF = percent body fat; BM = body mass; EA = energy availability; EI = energy intake; FFM = fat-free mass; FM = fat mass; SMM = skeletal muscle mass; *n* = 13; * *p* < 0.05 based on Pearson correlational analysis.

## Data Availability

Data pertaining to this study can be made available upon request.
